# Learning Beliefs, Time on Platform, and Academic Performance During the COVID-19 in University STEM Students

**DOI:** 10.3389/fpsyg.2021.780852

**Published:** 2021-12-16

**Authors:** Karla Lobos, Fabiola Sáez-Delgado, Rubia Cobo-Rendón, Javier Mella Norambuena, Alejandra Maldonado Trapp, Nataly Cisternas San Martín, Carola Bruna Jofré

**Affiliations:** ^1^Laboratorio de Investigación e Innovación educativa Dirección de Docencia, Universidad de Concepción, Concepción, Chile; ^2^Centro de Investigación en Educación y Desarrollo (CIEDE-UCSC), Departamento Fundamentos de la Pedagogía, Facultad de Educación, Universidad de Católica de la Santísima Concepción, Concepción, Chile; ^3^Programa de Doctorado Educación en Consorcio, Universidad de Católica de la Santísima Concepción, Concepción, Chile; ^4^Departamento de Física, Facultad de Ciencias Físicas y Matemáticas, Universidad de Concepción, Concepción, Chile; ^5^Departamento de Bioquímica y Biología Molecular, Facultad de Ciencias Biológicas, Universidad de Concepción, Concepción, Chile

**Keywords:** learning beliefs, learning analytics, university students, higher education, COVID-19

## Abstract

Due to the closure of universities worldwide because of the COVID-19 pandemic, teaching methods were suddenly transformed to an emergency remote teaching (ERT) modality. Due to the practical nature of STEM courses, students cannot participate in activities in which manipulating objects is necessary for accomplishing learning objectives. In this study, we analyze the relation among STEM students learning beliefs at the beginning of ERT (T1) with their Learning Management systems (LMS) time-on-task and their final academic performance (T2) during the first semester of ERT. We used a prospective longitudinal design. 2063 students (32.3% females) from a university in Chile participated, where the academic year starts in March and finishes in December 2020. We assessed their learning and performance beliefs through an online questionnaire answered at the beginning of the academic period (T1). Then, using learning analytics, time invested in the CANVAS LMS and the academic performance achieved by students at the end of the semester (T2) were assessed. The results show that students mainly stated negative beliefs about learning opportunities during ERT (*n* = 1,396; 67.7%). In addition, 48.5% (*n* = 1,000) of students stated beliefs of “medium” academic performance for the first semester (T1). Students with lower learning beliefs at T1 spent less time in the LMS during the semester and had a lower academic performance at T2 than students who had higher learning beliefs at T1. The implications of these findings on the role of instructors and institutions of higher education are discussed.

## Introduction

It is expected that the demand for professionals in STEM careers will increase in the coming years (Avendaño Rodríguez and Magaña Medina, 2018; UNESCO, 2019). Furthermore, the World Economic Forum held in 2020 revealed that critical thinking and collaboration are highly demanded competencies and that this need will keep increasing. These aspects, along with other skills, such as foundation literacy and character qualities, are 21st-century skills. Developing these abilities in students and professionals implies the need for implementing active learning teaching strategies in STEM areas (Soler and Dadlani, 2020). Active learning strategies consider students the main responsible for their education by realizing meaningful activities in which the teacher acts as a facilitator or guide during the learning process (Hernández-De-Menéndez et al., 2019).

In this context, research on how to improve STEM students’ teaching and learning is relevant (Hou et al., 2021). However, today, STEM students have undergone a transformation in their learning experiences due to the COVID-19 pandemic (Cannon et al., 2021). The sudden change of teaching modality that all students worldwide suffered due to the pandemic is known as emergency remote teaching (ERT; Bozkurt and Sharma, 2020; Bustamante, 2020). This denomination is because the conditions of online education created during COVID-19 were not planned as expected in other scenarios (Pappas and Giannakos, 2021). Therefore, the improvisation and ingenuity of many instructors, who were not prepared for such a drastic change of teaching modality, prevailed (Hodges et al., 2020; Lobos Peña et al., 2021).

Studies conducted during the ERT period identified that: (a) students fear facing many difficulties while working online and believe that their instructors could not help them enough (Akcil and Bastas, 2020), (b) students show higher motivation for online learning when they perceived greater usefulness and ease in virtual learning tools, so the thoroughness in the choice and planning of resources and activities is crucial (Cicha et al., 2021), and (c) students were more satisfied with online education when they perceived less impact of the pandemic on the preparation and adaptation to the virtual format of their educational institutions (Gonçalves et al., 2020). As valuable as the ERT is, accelerating the implementation of teaching processes caused the loss of several critical elements for its effectiveness (Hodges et al., 2020). Therefore, if not guaranteed, the ERT leads to a modality in which the aim is to replicate face-to-face strategies instead of taking advantage of the resources and benefits of online learning systems. The effectiveness of online education lies heavily in the careful design and preparation of learning resources, activities, and assessments following an instructional design that is appropriate to the course.

### Learning Beliefs

Self-efficacy beliefs in the context of online learning refer to students’ beliefs regarding being able to execute, successfully, the tasks and activities presented in the virtual learning environment (Cai et al., 2017; Al-Rahmi et al., 2018); for example, believing in their ability to use the learning management system of their institution. In addition, these technological tools respond to educational processes and the capacity to deploy self-regulated learning that requires greater autonomy, clear goals, among other capabilities involved (Carter et al., 2020; Qetesh et al., 2020). In an online learning context, students report low perceptions of learning (Chen et al., 2018) and low levels of academic self-efficacy (Casanova et al., 2018; Gopal et al., 2021) when the courses do not follow an instructional design specifically created for online courses.

Systematic reviews of the literature indicate that beliefs about student learning can impact academic performance and dropout rates (Richardson et al., 2012; Honicke and Jaclyn, 2016). If self-efficacy is low, student engagement and performance will be low (Van der Houwen et al., 2010; Valle et al., 2015; Borzone, 2017), whereas dropout intention will be higher (Casanova et al., 2018). In the context of the pandemic, instructors and students expressed low learning beliefs about virtual education at the beginning of the academic period in two longitudinal investigations. As a result, students had little confidence in online education’s opportunities regarding the quality of teaching processes, learning materials and activities, and collaborative work with peers and instructors (Camfield et al., 2021; Lobos Peña et al., 2021). Conversely, students obtain better academic performance and are more satisfied with the teaching and learning processes when their learning beliefs are higher (Kostagiolas et al., 2019).

Research of students’ self-efficacy beliefs and behavior in online education is incipient. Despite this, there are already few reports indicating that students who believe they will perform better in an online modality will interact more with learning activities and resources in virtual environments (Ifenthaler, 2020; Ifenthaler and Yau, 2020). In this same area, self-efficacy beliefs have been specified around academic achievement in technology-mediated learning experiences.

### Learning Analytics: Time on Platform

Learning analytics is defined as the process of measuring, collecting, analyzing, and reporting data about learners and their contexts to promote learning by considering elements, such as data, data analysis, and intervention measures generated from them (Romero and Ventura, 2020). Concerning the students, the use of analytics allows the integration of information, such as their behavior during the teaching and learning process, their past or current academic performance, sociodemographic information, among others (Zilvinskis and Willis, 2019). These data allow for statistical analysis and predictive models that facilitate the early detection of students at possible risk of failure (Larrabee Sønderlund et al., 2019). Furthermore, the user can predict learners’ success during a course using various performance indicators with learning analytics. For example, you can use grades from previous courses or learners’ current performance. Tracking learner activity in the LMS is also commonly used (Liz-Domínguez et al., 2019).

One of the most studied variables in learning analytics research is the platform time or *time-on-task* invested by students during online education (Ifenthaler and Yau, 2020). For example, considering when an event starts and ends is especially important when defining how long students are actively working in the LMS. The opportunity to extract this kind of information makes learning analytics data to be considered as the digital footprint left by students in the context of an online course, as it allows to estimate the level of involvement and the effort they deploy during their courses (Miller and Soh, 2013; Rojas-Castro, 2017). Moreover, research in the ERT period has indicated that students who believe they will do better in an online modality interact more with virtual environments’ learning activities and resources (Ifenthaler, 2020; Ifenthaler and Yau, 2020). So, reviewing learning analytics becomes relevant for those seeking to develop intentional pedagogical actions to improve educational outcomes. Nevertheless, it is essential to know and analyze the student’s interaction with the resources and activities, connection times, and connection moments. These will allow us to understand the final performance better and address low performances or behaviors that lead to it (Zhang et al., 2020).

After the pandemic, universities will likely employ blended learning, which considers quality training with specially designed virtual teaching environments linked to face-to-face teaching to enhance students’ educational experience by responding to their needs (McGrath et al., 2021). A blended learning modality will have to incorporate all the knowledge developed by instructors and institutions during the pandemic regarding virtual tools and mix them with the best practices of face-to-face classrooms. Unfortunately, this kind of modality has been scarce in Latin America. Therefore, this paper aims to contribute knowledge supporting virtual tools in STEM undergraduate programs in higher education. In this sense, our objective is to evaluate the relation among STEM students learning beliefs at the beginning of the ERT (T1) with their LMS time-on-task during the first semester of the ERT and their final academic performance (T2).

With this research, our goals are:

1. Describe the learning beliefs of STEM undergraduate students during the COVID-19 pandemic ERT at the beginning of the academic semester (T1).
2. Identify the interaction level with the LMS Canvas after the end of the academic semester (T2) of STEM university students during the ERT
3. Compare the connection time of STEM university students considering variables, such as gender and academic level to which they belong.
4. Analyze the learning beliefs of undergraduate students in the STEM area (T1), considering the student’s interaction with the LMS and the academic performance achieved at the end of the semester (T2).

## Materials and Methods

**Figure 1:**
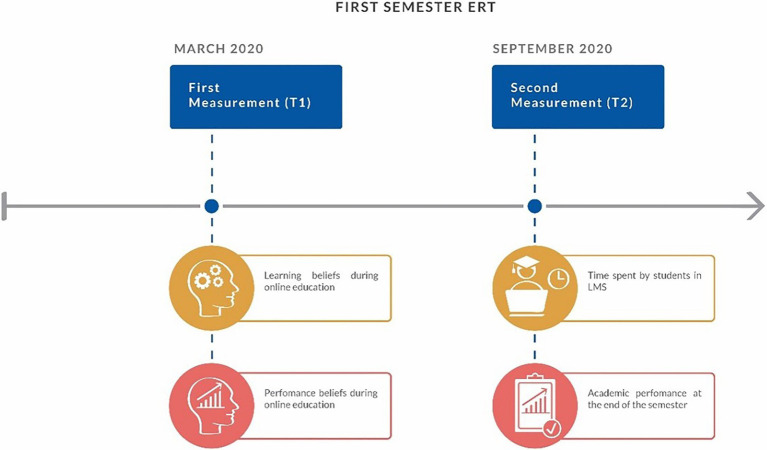
Description of the measurement moments carried out in the investigation.

The method used in this research was in the framework of a simple prospective design (Ato et al., 2013). In Figure 1, we describe the measurement timeline.

### Participants

Participants were 2063 undergraduate STEM students from a university in Chile, where the academic year starts in March and finishes in December 2020. Gender distribution was 32.3% (664) females and 67.8% (1399) males. The average age was 21.31 years (*SD* = 2.64). The distribution according to STEM areas was: 1485 students from Engineering (71.9%), 315 students from Physical Sciences and Mathematics (15.3%), 185 from Chemical Sciences (9.0%), and 78 students from Biological Sciences (3.8%). Concerning academic level, 641 were 1st-year students (31.1%), and 1,422 were students in higher courses (68.9%).

### Measurement Instruments

#### Learning Beliefs

The institution developed and massively implemented a two-item survey at the beginning of the academic period during the ERT due to the COVID-19 pandemic (T1): (1) I think my learning opportunities in online learning will be, and (2) I think my academic performance will be. The first item had two possible response options (1 = worse than in face-to-face learning, 2 = the same as in face-to-face learning), whereas the second item had three options (1 = low, 2 = medium, 3 = high). Therefore, when we talk about learning beliefs, we refer to the student’s beliefs about their opportunities to learn and maintain their academic performance during ERT due to COVID-19.

#### Time on Learning Management Systems

The institution analyzed the students’ time-on-task during the ERT semester. We defined this variable as the time between two interactions (or events associated with a timestamp) LMS CANVAS, with a 10-min threshold. If the user did not perform any action by 10 min, the session is considered finished. Our definition of the time-on-task threshold was based on the evidence described in the literature (Kovanović et al., 2015) and on our researchers’ experience. It is important to note that the ways to determine time-on-task in LMS are still under investigation because it depends on the context and characteristics of the data (Godwin et al., 2016).

#### Academic Performance

We measured participants’ grade point average by the average grade obtained over the first semester during the ERT. Each faculty provided this information from the institutional records. In Chile, the grading system is constructed on a scale from 1.0 to 7.0 points. The grades from 6.0 to 7.0 correspond to an academic performance considered as “excellent.” The grades from 5.0 to 5.9 are labeled as “good” grades, while 4.0–4.9 are defined as “satisfactory.” Last, grades from 1.0 to 3.9 are “unsatisfactory,” which means the student failed the course (MINEDUC, 2020).

### Procedure

This research was approved by the Ethics Committee of the participating university, confirming the ethical criteria for research with human beings. The informed consent form was presented, describing research goals and characteristics for participation in the study.

The questions on learning beliefs were part of a general questionnaire applied in digital format and sent to the students’ institutional e-mails. The reception of responses to this questionnaire was at the beginning of the academic year, during March 2020 (T1). LMS CANVAS platform (John, 2021) supplies the proportion of time in the virtual classroom. In addition, each faculty provide academic performance from the institutional records. We measured these two variables at the end of the first academic semester, during September 2020 (T2).

Assumptions of normality of the data were checked using the Kolmogorov–Smirnov test with Lilliefors modification (Thode, 2002). We applied the Levene test (Fox and Weisberg, 2018) to verify the constant variance between groups (homoscedasticity). Due to the non-normality of data, presence of outliers, and in some cases, absence of homoscedasticity, we performed Yuen’s test (Yuen, 1974) for the comparison of two groups and one-way ANOVA test for trimmed means (Wilcox and Tian, 2011) for statistical analyses employing more than two groups. The method proposed by Algina et al. (2005) was employed for the effect size analysis of the results. For data analysis, we used RStudio software version 4.0.3 (2020-10-10).

## Results

The goal of this study was to analyze learning beliefs during online education (T1) and their link with the time invested by students in the LMS and with the academic performance achieved at the end of the semester (T2) in the context of the ERT 2020. The findings are presented below.

### STEM Students’ Beliefs About Online Learning During ERT Context

Regarding undergraduate STEM students’ learning beliefs during the ERT for the COVID-19 pandemic (T1), students mainly stated negative beliefs about learning opportunities (*n* = 1,396; 67.7%). Only 32.3% (*n* = 667) of participants declared that they believed online education would provide them with good learning. When analyzing the characteristics of students according to their learning beliefs, we found that there were no statistically significant differences according to students’ gender and the type of school they came from (public, private, subsidized).

When assessing students’ beliefs about their academic performance in the ERT context, 48.5% (*n* = 1,000) of participants stated beliefs of “medium” academic performance for the first semester, 30.8% declared beliefs of “high” performance, and 20.7% (*n* = 427) of students stated that they would perform “poorly” in the ERT context. In the case of achievement beliefs, when analyzing the characteristics of the participants in each group, we found that students with higher achievement beliefs [*F*(2,637.7) = 6.09, *p* = 0.002] presented higher scores on the mathematics university entrance test or PSU, (*M* = 665.23; *SD* = 64.34) than students with lower achievement beliefs (*M* = 638.978; *SD* = 83.79).

### Connection Time in the Virtual Classroom (LMS) by Students During the ERT Context

Students spent an average of 92.87 h (*SD* = 81.59) in the virtual classroom during the entire semester (T2). The time spent by students on the platform was analyzed considering gender. Males spent an average of 94.17 h (*SD* = 85.23) in the virtual classroom, while women spent 90.13 h (*SD* = 73.35). When analyzing differences in the students’ connection time according to gender, the results were not statistically significant [*t*(820.19) = 0.0006, *p* = 0.999]. Therefore, there is no distinction in connection time in the LMS between men and women (see Table 1).

**Table 1:** Differences in students’ platform times as a function of student learning beliefs, gender, and academic year to which they belong.

	Beliefs about online learning during ERT		Yuen test		
Connection time to the LMS (hours)	Positive (*n* = 667)	Negative (*n* = 1,396)	*t*	*p*	Effect size
	102.83 (*SD* = 88.46)	88.11 (*SD* = 77.68)	T(690.66) = 3.43	<0.001	0.18
	Academic level							
	First-year (*n* = 641)	Upperclassmen (*n* = 1,422)	*t*	*p*	Effect size
	99.56 (*SD* = 81.23)	89.86 (*SD* = 81.62)	T(806.38) = 3.557	<0.001	0.17
	Gender							
	Women (*n* = 664)	Male (*n* = 1,399)	*t*	*p*	Effect size
	90.13 (*SD* = 73.35)	94.17 (*SD* = 85.23)	T(820.19) = 0.00	0.99	-

SD = standard deviation; n = number of participants.

**Figure 2:**
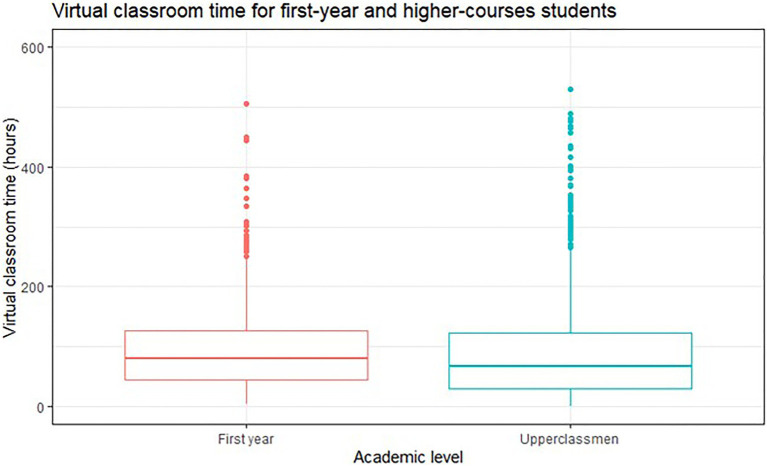
Distribution of students’ connection time to the Learning Management systems (LMS) during the emergency remote teaching (ERT) semester according to their academic level.

For the analysis of connection time to the LMS according to academic level (1st-year students regarding students taking second through 4th-year courses), we identified 641 (31.1%) freshmen and 1,422 (68.9%) students attending 2nd-year courses or higher. The 1st-year students spent an average of 99.56 h (*SD* = 81.23), whereas senior students 89.86 h (*SD* = 81.62). Statistically significant differences were found [*t*(806.38) = 3.557, *p* < 0.001; ES = 0.17] in connection time to virtual classroom according to the academic year of students (see Figure 2). In this case, 1st-year students presented a longer connection time in the LMS than the upper-course students.

### Students’ Learning Beliefs, Time Spent Online in the Virtual Classroom, and GPA

Learning beliefs were categorized into students who stated positive beliefs regarding their learning and students who declared negative beliefs. Students with positive learning beliefs accessed an average of 102.83 h (*SD* = 88.46) to the LMS. On the other hand, students with negative beliefs accessed an average of 88.11 (*SD* = 77.68) to the LMS. When evaluating connection time on the LMS, we found statistically significant differences between the groups [*t*(690.66) = 3.43, *p* < 0.001, ES = 0.18], observing that students with positive beliefs about learning spended more hours connected to the LMS (see Table 2).

**Table 2:** Differences in academic performance obtained by participating students at the end of the semester (T2) as a function of performance beliefs during the ERT (T1).

	Academic performance beliefs			One-way ANOVA on trimmed means		
	High (*n* = 636)	Medium (*n* = 1,000)	Low (*n* = 427)	*F*	*p*	Effect size
Final grade	5.78 (*SD* = 0.55)	5.72 (*SD* = 0.52)	5.61 (*SD* = 0.51)	F(2,633.45) = 9.50	<0.001	0.16

SD = standard deviation; n = number of participants.

**Figure 3:**
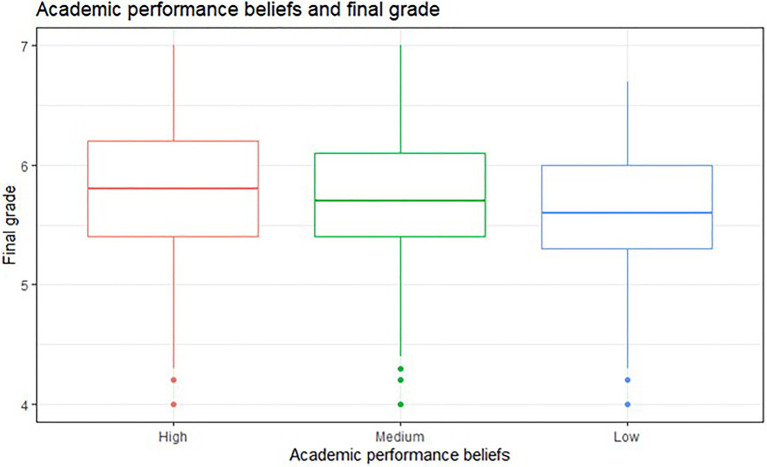
Description of academic performance beliefs (T1) and final grade (T2) from STEM students’ during the ERT semester.

We assessed the differences between students’ academic performance (T2) and their beliefs about academic performance at the beginning of the semester (T1). Students were organized into three performance belief groups (low, medium, high). Participants who stated “low” performance beliefs obtained an average grade of 5.61 (*SD* = 0.51), while students who declared “medium” performance beliefs obtained on average a mark of 5.72 (*SD* = 0.52). Finally, students stating “high” academic performance beliefs scored on average 5.78 (*SD* = 0.55). We found statistically significant differences between the groups [*F*(2,633.45) = 9.4984, *p* < 0.001]. Students with “low” performance beliefs (T1) obtained lower grades at the end of the semester (T2) than students with “high” (*p* < 0.001) and “medium” performance beliefs (*p* < 0.01; see Figure 3).

## Discussion

Due to the COVID-19 pandemic, university students around the world had to continue their training remotely. The ERT is characterized as unplanned and temporary since its implementation is associated with an emergency. Therefore, neither educational institutions, students, nor instructors were prepared to carry out educational processes efficiently in this context. This situation had an impact on the students’ experiences of their university education. However, it was particularly detrimental to students of majors in STEM due to the practical nature of the courses.

We identified the relationship between the following three variables: learning beliefs, time spent on tasks on the LMS, and academic performance achieved at the end of the semester.

### STEM Students’ Beliefs About Online Learning During ERT Context

As a result of the present study, more than half of the participants had negative beliefs about online learning during the ERT context. In the published empirical evidence, mixed results were found concerning beliefs about online learning in STEM. For example, an investigation of pharmacy students’ experience during the COVID-19 pandemic indicated that 49% of the participants showed a positive attitude toward online learning, and only 34% of the students identified barriers to online learning (Shawaqfeh et al., 2020). Another research with students from various areas of basic sciences found that participants perceived positive online learning experiences and considered that the situation was handled adequately (Almusharraf and Khahro, 2020). However, contrary to the results presented above, another research reports that although students state that online education is a modality responding positively to their needs, they express concerns regarding pedagogical, logistical, and administrative support from their institutions, negatively impacting their beliefs. Moreover, students state that it is difficult to connect with their professors and classmates (Katz et al., 2021; Rivera-Vargas et al., 2021). These findings agree with the results of this study.

In our context, from October 2019 to February 2020, a social movement developed in Chile due to citizens’ discontent with the government (Morales Quiroga, 2020). Strikes and marches characterized this movement within the second semester of the 2019 academic year. In this period, educational institutions had to implement the ERT modality due to the social situation. After finishing the second semester of 2019 in ERT modality due to the social movement, students started the first semester of 2020 in ERT modality due to the COVID-19 pandemic (Brunner et al., 2020). In the case of the university students in this research, we believe that the online education experience during the social movement accentuated negative beliefs.

To identify the ERT effects of the COVID-19 pandemic, we differentiated between two groups of STEM students: 1st-year students in 2020 and upper-level students. On the one hand, the latter group of students had a traditional learning experience that allowed them to participate in face-to-face cultural activities, meet peers, and engage in STEM activities, such as laboratory practices. On the other hand, the ERT’s effects on the 1st-year students are possibly higher since they did not have face-to-face experiences and all their training has been remote. Concerning academic performance beliefs, a significant number of students stated that they could achieve medium to high performance during the first semester of 2020, i.e., the first ERT semester. This result is similar to one reported by students in other research where they indicate beliefs of having positive or closer to expected results in the ERT scenario (Rager, 2020). This finding could be associated with personal factors of the student, for example, the young people’s level of commitment to their university undergraduate programs, and with their self-efficacy beliefs about completing academic assignments from a digital modality, the use of social support sources (peers and family) and the technological resources available to them for the implementation of academic activities (internet or computers).

In the ERT scenario, the student’s socio-academic integration process was significantly transformed. Their institutional experiences were developed from virtuality, limiting the development of the young people’s academic identity. According to Tinto’s theory, students’ success resides in their ability to integrate socially and academically into the university (Tinto, 1975). This model proposes that students see themselves as part of the educational institution when they can frequently interact with peers, instructors, and the university community. This process increases their commitment to the career, benefiting their academic performance and persistence (Tinto, 2017). In this regard, universities should consider implementing programs and policies that benefit the social integration of students during the period of return to higher education institutions due to the control of the COVID-19 pandemic.

### Connection Time in the Virtual Classroom (LMS) by Students During the ERT Context

Regarding students’ time-on-task in the LMS, we analyzed students, interaction with the platform in their courses in ERT modality. This variable was selected to better understand the students’ learning process during this period (Klašnja-Milićević et al., 2017). Our results show that, on average, 1st-year students logged in to the LMS longer than students in higher courses.

Considering that 1st-year students present a higher dropout risk (Bernardo et al., 2015, 2016), we believe these results can be considered positive, especially in STEM students (Jungert et al., 2019). Still, the low perception of learning opportunities and low platform time of STEM students may exacerbate the dropout figures that already existed before the pandemic (Van den Hurk et al., 2019). For such reason, there is a need to strengthen student engagement in the LMS coupled with student satisfaction with the study content and experience (Fleischer et al., 2019). Furthermore, the students’ difficulties during the ERT could explain the low beliefs regarding learning opportunities in this period. For this reason, university authorities must reinforce and maintain the mechanisms of consultation and accompaniment for the implementation of online learning.

### Students’ Initial Learning Beliefs, Time Spent Online in the Virtual Classroom, and GPA

Likewise, we found that low beliefs about learning opportunities and academic performance are related to lower time-on-task and lower academic performance achieved at the end of the semester. This result can be explained from the theoretical approach of student self-efficacy. When students have low beliefs or perceptions of learning ability, they present difficulties regarding their academic performance (Bandura, 2012). In the case of the online setting, when students possess positive beliefs toward learning experiences, they interact to a greater extent with the learning activities and resources in the LMS, leading to higher performance (Ifenthaler, 2020).

Participating students had lower expectations about their learning opportunities, although they believed that the ERT would not affect their academic performance. Based on these findings, research indicates that individual and educational factors affect students’ beliefs about virtual learning (Alameri et al., 2020). In this case, uncertainty about how the ERT would unfold, being a new learning experience for instructors and students may have impacted students’ beliefs about their learning opportunities. Therefore, we did not identify significant changes in students’ performance expectations in the ERT. However, Redondo-Gutiérrez et al. (2017) found that when students’ performance expectations relate with taking individual assessments, they tend to be better than in the case of group assessments (Redondo-Gutiérrez et al., 2017). Regarding assessment during the ERT, students were not clear about the evaluative processes of their learning. One explanation for this result could be that the students believed that assessment processes would be individual due to the ERT. As a result, they believed that their grade would be the result of their performance.

Although institutions’ instructional designers and managers understand the difference between ERT and online education, students and instructors usually do not (Chaka, 2020). Only conducting video lectures or leaving the material in repositories is far from representing a successful online educational model. In online education courses, planning and design must follow an instructional model, such as ADDIE or Backward Design (Dean, 2019; Ofosu-Asare et al., 2019). Each learning activity and resource is carefully planned to be implemented through an LMS. Nonetheless, we know that it was necessary to improvise in the ERT period to provide continuity to the educational processes, which resulted in inadequate preparation for both students and instructors. In addition, the uncertainty generated by the crisis could also be reflected in students’ negative beliefs about online learning.

Particularly in STEM courses, the perception of low learning may be influenced by the disciplines’ characteristics. Usually, students learn to implement the scientific practices through hands-on educational experiences in face-to-face laboratories, especially in chemistry, physics, and biology. Although scientific practices can be taught through academic activities, some require students to touch and manipulate elements and instant interaction with their peers. For example, in physics courses, students should learn to manipulate oscilloscopes; in chemistry courses, they should learn to manipulate reagents; and in biology, they should learn to manipulate microscopes. On the other hand, there are exceptions in which the implementation of virtual rather than face-to-face laboratories should not cause negative effects. An example of this is the computer science courses, in which the laboratories were already performed employing computers, using programming languages, such as Python, R, or Fortran. However, it should not be forgotten that such activities existed before the ERT caused by COVID-19 pandemic. The success in using these instructional strategies, whether in the face-to-face or online modality, lies in the importance of linking it with the possibility of teamwork and the realization of adequate feedback by the teacher on the student’s learning.

Given the impossibility of implementing face-to-face laboratory activities for all students, university instructors in STEM faced additional challenges compared to other disciplines. Instructors had to confront an extra challenge to support their students in achieving course learning outcomes: looking for a solution to implement laboratory practices remotely and incorporating active learning techniques (Vogel-Heuser et al., 2020). An option would be implementing simulation-based virtual laboratories where students must modify parameters to see the effects in the experiments (Belford and Moore, 2016). An excellent resource to use is the University of Colorado Boulder’s repository of interactive simulations. Simulations include activity proposals containing the HTML iframe code to embed the simulation directly into the LMS (University Colorado Boulder, 2021). Additionally, there are different alternatives to remote laboratories. An example of these is an educational project of the University of Deusto (Orduña et al., 2011; García-Zubía et al., 2018; Orduña et al., 2018).

Another remedial action could be implementing a peer support system by upper-level training students with high learning beliefs to support 1st-year students with low beliefs in online learning processes (Honkimaki and Tynjala, 2018). Moreover, institutions could also implement a remote help desk system where students can send their queries related to ERT. For the latter initiative, the institution needs access to help desk software and staff trained in pedagogical and technological aspects to provide timely and efficient student responses.

A strength of this research is the use of other forms of measurement to reduce the possible bias that could be generated by only using the self-report as a measurement (De las Cuevas Catresana and González de Rivera, 1992). In this study, the application of questionnaires allowed to assess young people’s learning and performance beliefs. Also, through learning analytics, we evaluated students’ behavior within the LMS during the entire semester and linked it with the academic performance achieved at the end of the period. Employment of learning analytics to assess students’ beliefs, especially in 1st-year students, enables early identification of students at dropout risk and provides personalized support before the student withdraws from the university (Honkimaki and Tynjala, 2018; Wong et al., 2018).

This study has some limitations, which are referred to the following aspects: (a) the presence of biases against the actual assessment of students’ academic performance, since due to the COVID-19 pandemic, the participating university implemented a series of educational policies that could affect the academic results obtained by the students; (b) the participants in this study belong to a single university, which, although it is one of the largest institutions of higher education in Chile, and with a great variety of disciplines, presents its own contextual characteristics that could affect the results; (c) the use of a single indicator (time-on-task) for the construction of the learning analytics variable is insufficient to cover the variety of behaviors that characterize the student’s interaction with the virtual classroom; (d) The measurement of the learning beliefs variable with two items could limit the content validity; and (e) finally, in this investigation it was not possible to identify other variables that could impact the time spent by students in the LMS, such as course design, number of credit hours, among others.

Future research could consider other elements, such as participation in forums, number of activities performed in the LMS, number of resources read and downloaded, among other analytics offered by the LMS (Ifenthaler and Yau, 2020). Also, it would be interesting to study further the effects of course variables (e.g., level, type, design, credit hours), which could impact student academic performance. Investigation along these lines could expand and diversify the sample and conduct similar studies when the pandemic context is overcomed.

This study contributes to the early identification of at-risk students, encouraging pedagogical actions to decrease students’ negative beliefs about online learning. In addition, our results could positively impact the dropout rates found in STEM careers by guiding institutional actions that address beliefs toward online education. These actions are significant given that post-pandemic, it is expected that a large part of Higher Education institutions seeks to promote Blended Learning education within their educational models. All the above consider technological advances, globalization of information, and learning about online education generated during the ERT.

## Conclusion

Considering the findings, we concluded that most students had negative beliefs about their opportunities to learn through the ERT. These beliefs were equally presented among men and women. We identified that students in their first academic year spended more time connected to the LMS. Additionally, we observed that when students presented positive beliefs about their learning, they spent more hours connected to the LMS.

We found that students with higher achievement beliefs presented higher scores on the mathematics college entrance test (PSU). Thus, we believe that the PSU score intervened in students’ future performance beliefs. Similarly, we identified that students with low-performance beliefs at the beginning of the ERT presented lower scores at the end of the ERT semester. The students’ beliefs about learning opportunities and performance intervened in the time of interaction with the LMS, affecting the academic achievement. Thus, it is relevant for teachers and institutions to promote beliefs that can relate to positive behaviors in their students.

## Data Availability Statement

The raw data supporting the conclusions of this article will be made available by the authors, without undue reservation.

## Ethics Statement

The studies involving human participants were reviewed and approved by Universidad de Concepción Ethics Committee. The patients/participants provided their written informed consent to participate in this study.

## Author Contributions

KL and FS-D contributed to the design of the study, literature, and writing of the manuscript. RC-R contributed to the design of the study, data analysis, and review of the abstract and manuscript. JM contributed to the data extraction, data analysis, and interpretation of the results. AM contributed to the study’s design, interpretation of the results, and the abstract and manuscript review. CB and NC contributed to the interpretation of the results and writing of the manuscript. All authors contributed to the article and approved the submitted version.

## Funding

Research reported in this publication was supported by Unidad de Fortalecimiento Institucional of the Ministerio de Educación Chile, project InES 2018 UCO1808 Laboratorio de Innovación educativa basada en investigación para el fortalecimiento de los aprendizajes de ciencias básicas en la Universidad de Concepción.

## Conflict of Interest

The authors declare that the research was conducted in the absence of any commercial or financial relationships that could be construed as a potential conflict of interest.

## Publisher’s Note

All claims expressed in this article are solely those of the authors and do not necessarily represent those of their affiliated organizations, or those of the publisher, the editors and the reviewers. Any product that may be evaluated in this article, or claim that may be made by its manufacturer, is not guaranteed or endorsed by the publisher.
